# RNA Interference-Mediated Knockdown of *Bombyx mori* Haemocyte-Specific Cathepsin L (*Cat L*)-Like Cysteine Protease Gene Increases *Bacillus thuringiensis kurstaki* Toxicity and Reproduction in Insect Cadavers

**DOI:** 10.3390/toxins14060394

**Published:** 2022-06-08

**Authors:** Linlin Yang, Yanyan Sun, Meiling Chang, Yun Zhang, Huili Qiao, Siliang Huang, Yunchao Kan, Lunguang Yao, Dandan Li, Camilo Ayra-Pardo

**Affiliations:** 1 China-UK-NYNU-RRES Joint Laboratory of Insect Biology, Henan Key Laboratory of Insect Biology in Funiu Mountain, School of Life Sciences and Agricultural Engineering, Nanyang Normal University (NYNU), Nanyang 473061, China; yanglinlin202205@163.com (L.Y.); yanyansun565340120@163.com (Y.S.); changmeiling2022@163.com (M.C.); zy174812@163.com (Y.Z.); qhlonline@hotmail.com (H.Q.); silianghuang@aliyun.com (S.H.); kanyunchao@163.com (Y.K.); lunguangyao@163.com (L.Y.); 2 School of Life Science, Henan University, Jin Ming Avenue, Kaifeng 475004, China

**Keywords:** insect pathogen, insect immunity, RNAi, gene function, bacterial clearance

## Abstract

The silkworm’s *Cat L*-like gene, which encodes a lysosomal cathepsin L-like cysteine protease, is thought to be part of the insect’s innate immunity via an as-yet-undetermined mechanism. Assuming that the primary function of Cat L-like is microbial degradation in mature phagosomes, we hypothesise that the suppression of the *Cat L*-like gene expression would increase *Bacillus thuringiensis* (*Bt*) bacteraemia and toxicity in knockdown insects. Here, we performed a functional analysis of *Cat L*-like in larvae that were fed mulberry leaves contaminated with a commercial biopesticide formulation based on *Bt kurstaki* (*Btk*) (i.e., Dipel) to investigate its role in insect defence against a known entomopathogen. Exposure to sublethal doses of Dipel resulted in overexpression of the *Cat L*-like gene in insect haemolymph 24 and 48 h after exposure. RNA interference (RNAi)-mediated suppression of *Cat L*-like expression significantly increased the toxicity of Dipel to exposed larvae. Moreover, *Btk* replication was higher in RNAi insects, suggesting that Cat L-like cathepsin may be involved in a bacterial killing mechanism of haemocytes. Finally, our results confirm that Cat L-like protease is part of the antimicrobial defence of insects and suggest that it could be used as a target to increase the insecticidal efficacy of *Bt*-based biopesticides.

## 1. Introduction

The bacterium *Bacillus thuringiensis* (*Bt*) is a well-known insect pathogen with an extensive record as a successful biopesticide [1,2]. The killing pathway of *Bt* can be summarised as follows: after ingestion by susceptible insects, *Bt* uses a combination of highly specific pore-forming toxins and other virulence factors—including phospholipases, metalloproteases, and collagenases—to breach the gut barrier and enter the host’s body cavity, where it feeds on the nutrient-rich haemolymph to reproduce [3,4]. The *Bt*-infected insects eventually die of acute septicaemia. Nevertheless, insects have a chance of survival after *Bt* infection. Immune responses to sublethal doses of *Bt* have been found to reduce bacterial pathogenicity, which may accelerate the development of resistance [5,6,7,8,9]. Identifying insect factors that interfere with *Bt* pathogenesis may help to develop new formulations with increased toxicity.

Insect innate immunity comprises humoral and cellular responses [10]. Humoral responses involve the melanisation and secretion of antimicrobial peptides (AMPs) and complement proteins [11], while cellular responses include phagocytosis and encapsulation of intruders by haemocytes (granulocytes and plasmatocytes) [12,13]. Transcriptomics of immunostimulated haemocytes has also revealed the complexity of the response, including activation of known immunity-related genes and other less characterised genes [14,15]. A novel haemocyte-specific *Cat L*-like gene, encoding a cathepsin L-like lysosome protease, was overexpressed in the haemolymph of silkworm larvae (*Bombyx mori*), injected with inactivated bacteria (*Pseudomonas aeruginosa* or *Staphylococcus aureus*) and pathogen-associated molecular patterns (PAMPs), such as peptidoglycan (from *Bacillus subtilis*) or lipopolysaccharides (from *Escherichia coli*) [16]. The authors suggest a possible role of this enzyme in innate immunity. So far, at least four classes of silkworm cathepsins have been described, namely B, D, O, and L, which are mainly involved in processes related to tissue remodelling during metamorphosis and response to a bacterial stimulus [17,18,19]. Assuming that the primary function of lysosomal Cat L-like cathepsin is microbial degradation in mature phagosomes, we hypothesise that the suppression of the *Cat L*-like gene expression would increase *Bt* bacteraemia and toxicity in knockdown insects. Previously, immunosuppressed larvae of *Spodoptera littoralis* were more susceptible to *Bt* in bioassays after *102Sl*—a gene involved in the control of encapsulation and nodulation responses—was silenced by RNA interference (RNAi) [20,21].

The present study aimed to test whether the RNAi-mediated suppression of the silkworm *Cat L*-like gene expression increases the toxicity of a *Bt* var. *kurstaki* (*Btk*)-based commercial formulation (Dipel) in insect bioassays. If Cat L-like is essential for bacterial clearing in silkworm haemolymph, the knockdown insects should be better suited for *Btk* proliferation.

## 2. Results

### 2.1. Cat L-Like Expression in Response to DiPel

**Figure 1:**
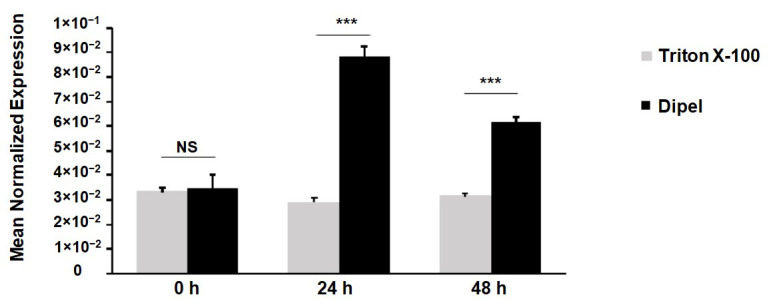
*Cat L*-like mRNA levels in response to a Dipel sublethal dose (2 μg/mL) determined by two-step qRT-PCR. Relative mRNA levels normalised to the reference gene *GAPDH* were expressed as ‘Mean Normalised Expression’ with Q-GENE. Each point represents the mean of three independent replicates ± SE. Significant differences were tested with Student’s *t*-test. *** *p* < 0.001; NS—not statistically significant.

The expression of the *Cat L*-like gene in Dipel-exposed silkworm larvae was analysed by quantitative polymerase chain reaction (qRT-PCR). First, we examined mortality data (LC_50_, the concentration causing death of 50% of the test population) of Dipel on one-day-old fifth instar larvae. Dipel showed strong larvicidal activity with an LC_50_ value of 2.88 ± 0.20 μg/mL (95% Fiducial Limits = 2.49—3.27 μg/mL; slope ± SE = 4.18 ± 0.24; *n* = 120 larvae) after 72 h of treatment. We then selected the Dipel dose of 2 μg/mL —a concentration below the LC_50_ for this larval stage— for the analysis of *Cat L*-like expression. Exposure of the insects to Dipel resulted in overexpression of the *Cat L*-like gene, measured at 24 h, *t* (4) = 18.82, *p* < 0.001, and 48 h, *t* (4) = 20.55, *p* < 0.001, after treatment (Figure 1).

### 2.2. RNAi of Cat L-Like Increased DiPel Toxicity

**Figure 2:**
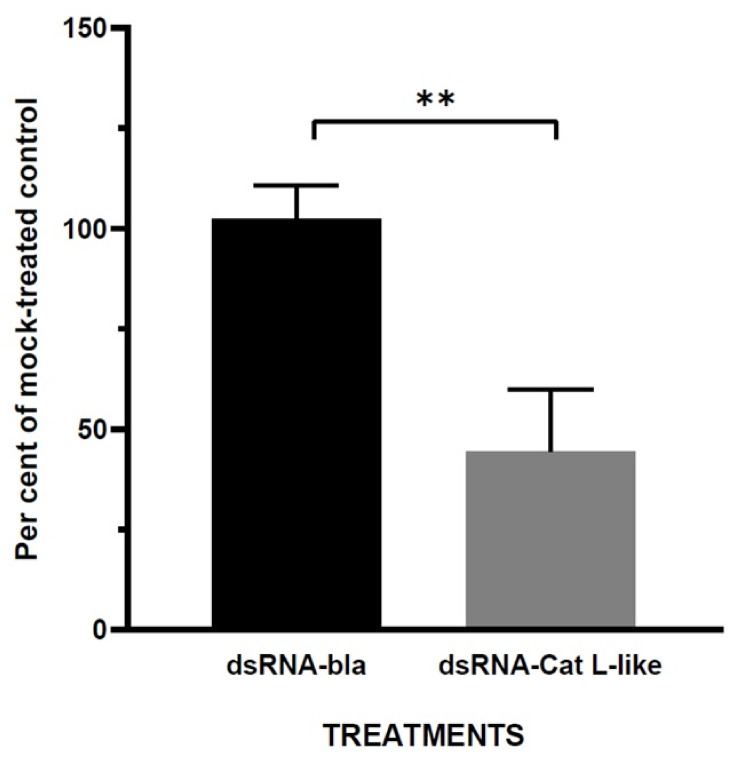
Reduction of *Cat L*-like mRNA transcription in haemocytes of Dipel-exposed silkworm larvae after RNAi treatment. Transcription levels of the *Cat L*-like gene were monitored by qRT-PCR in haemocytes from 5th instar larvae 48 h after injection of 2.5 μg dsRNA/larva of either dsRNA-Cat L-like or dsRNA-bla. Results are expressed as the per cent of mock-treated control (i.e., injected with water) after normalisation with the reference gene *GAPDH*. The bars represent the mean values of three independent replicates ± SE. ** *p* < 0.01.

Double-stranded RNA synthesised in vitro from a fragment of the *Cat L*-like gene (dsRNA-Cat L-like) was injected into one-day-old fifth instar larvae to induce insect RNA interference (RNAi) machinery prior to Dipel bioassays. Similar RNAi experiments, but using a fragment of the bacterial beta-lactamase (*bla*) gene (dsRNA-bla), were performed to detect possible off-target effects. A mock-treated control was produced by injecting the insects with water only. Injection of dsRNA-Cat L-like resulted in a significant (t (4) = 5.69, *p* < 0.01) reduction of *Cat L*-like transcripts in larval haemolymph of Dipel (2 μg/mL)-exposed insects after 12 h of exposure compared with non-specific dsRNA-bla (Figure 2); *Cat L*-like mRNA abundance decreased by more than 50% of the mock control. Moreover, injection of dsRNA-bla showed no significant effects on the *Cat L*-like target gene compared with the mock control. Furthermore, injection of dsRNAs did not result in visible morphological abnormalities in the pupae of insects not challenged with Dipel.

**Figure 3:**
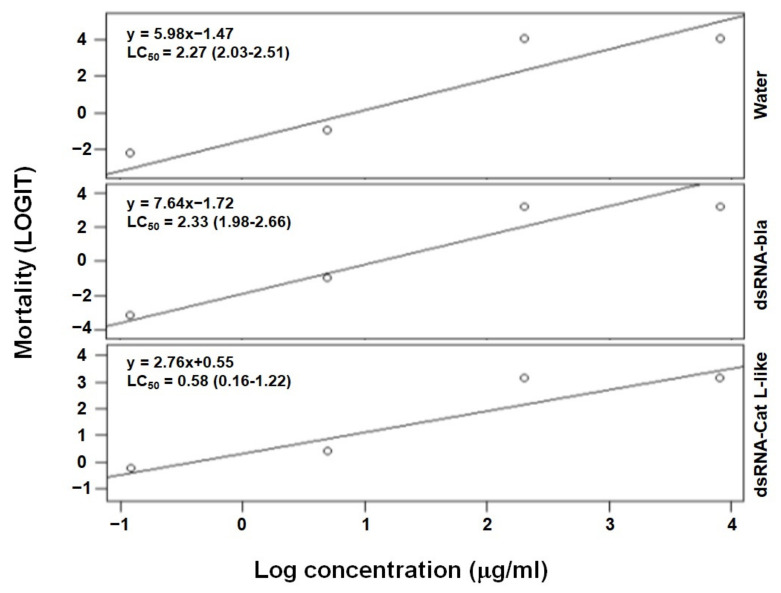
Mortality of silkworm larvae in bioassays with Dipel after RNAi of *Cat L*-like. Data points are from three-day mortality experiments. Lines represent minimal adequate fitted statistical models. Water and dsRNA-bla are control treatments; dsRNA-bla refers to a bacterial (non-specific) double-stranded RNA from the beta-lactamase (*bla*) gene to detect possible “off-target” effects of RNAi.

After RNAi, larvae were exposed to the same Dipel doses as in the bioassays and their survival was recorded. RNAi of *Cat L*-like significantly increased larval mortality compared to dsRNA-bla and mock controls (*p* < 0.01) (Figure 3). Furthermore, larval mortality rates did not differ in the non-specific dsRNA-bla and mock controls (*p* = 0.27), indicating that non-specific interactions with dsRNA did not affect the response to Dipel.

### 2.3. RNAi of Cat L-Like Enhances Btk Reproduction within Cadavers

**Figure 4:**
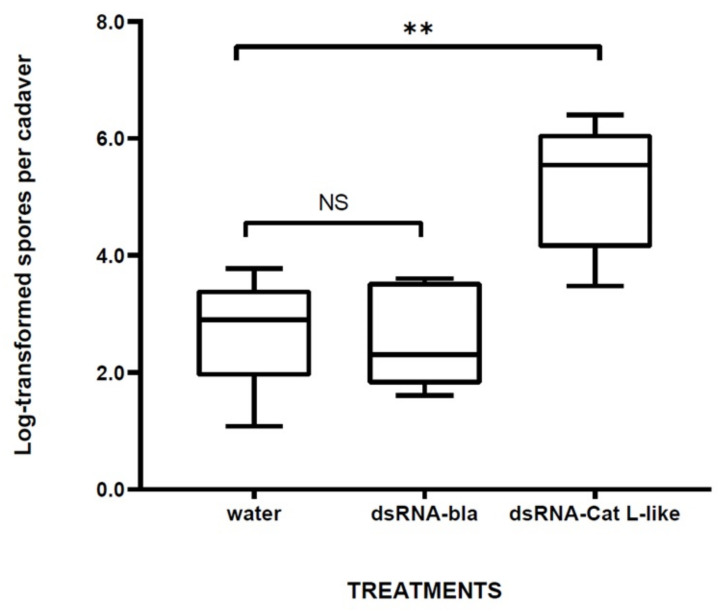
Relationship between *Btk* reproduction within the insect and RNAi treatment. Data are log-transformed spore counts per cadaver. Bars represent means ± SE. Analysis of variance (ANOVA) in conjunction with a post hoc Tukey–Kramer multiple comparison test was performed to determine differences among treatments. ** *p* < 0.01; NS—not statistically significant.

We further investigated the effects of Cat L-like RNAi on susceptibility to Dipel by analysing *Btk* replication in dead insects killed by Dipel. The number of spores per cadaver was significantly higher (*p* < 0.001) in dsRNA-Cat L-like compared with controls in insects exposed to a Dipel dose of 2 μg/mL (Figure 4). Cadavers of insects exposed to a lower Dipel dose (0.4 μg/mL) showed no *Btk* counts, while cadavers of insects exposed to higher Dipel concentrations (10 and 50 μg/mL) showed no significant differences in the number of *Btk* colonies counted between treated and controls (data not shown).

### 2.4. Phylogenetic Analysis of Cat L-Like Sequence

**Figure 5:**
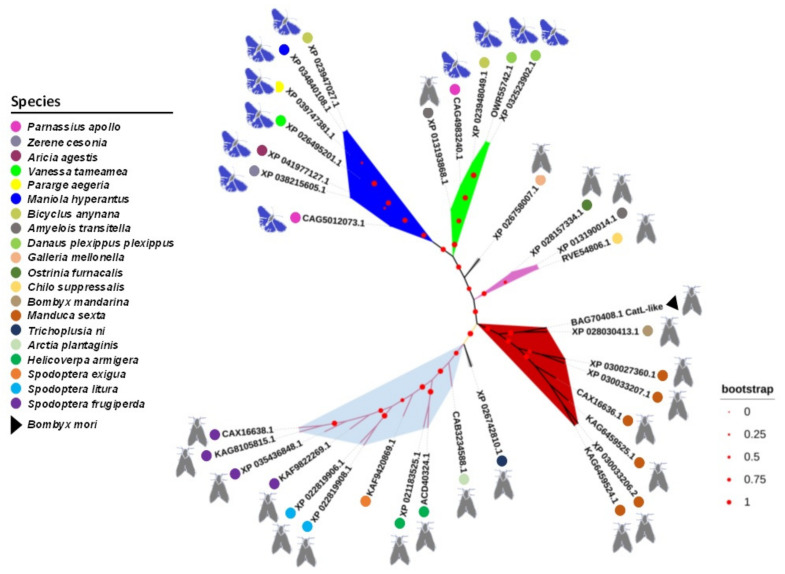
Unrooted tree showing the relationship between the silkworm Cat L-like cathepsin (BAG70408.1) and 34 selected homologous amino acid sequences of different moth and butterfly species (identified by their GenBank accession numbers) obtained with the iTOL v 6.5.4 programme. The sequences were phylogenetically aligned using the programme Muscle vs. 3.7. Bootstrap values (inferred from 1000 replicates) are given along the branches of the consensus tree.

Phylogenetic reconstruction of the Cat L-like predicted polypeptide and other 34 homologous Lepidoptera sequences from GenBank revealed that it was most similar to sequences from moth, not butterfly, species (Figure 5). In the unrooted tree, Cat L-like was in the same branch as sequences derived from the wild silk moth (*Bombyx mandarina*) and the sphinx moth (*Manduca sexta*), all of which belong to the same superfamily Bombycoidea.

## 3. Discussion

We have shown that the *Cat L*-like gene of the silkworm is induced in haemolymph after larvae are exposed to mulberry leaves contaminated with a sublethal dose of Dipel, possibly as part of the insect defence against the *Btk* bacterium. The *Btk* virulence factors in Dipel can rapidly degrade the intestinal mucosa of the poisoned insects [22], allowing the gut bacteria (*Btk* or symbiotic resident flora) to enter the body cavity and interact with the insect haemocytes in the haemolymph. Indeed, Caccia et al., (2016) described a similar microbiota in the haemocoel and intestinal lumen of *Bt*-poisoned *S. littoralis* larvae [20]. Previously, Dubovskiy et al., (2008) had shown that oral inoculation of the larvae of the greater wax moth (*Galleria mellonella*) with *Bt* increased cellular immune responses such as phagocytic activity and encapsulation rates in the insect haemolymph [23]. In silkworms, *Cat L*-like expression was found exclusively in spherulocyte and plasmatocyte cells [24]. The latter are known for their role in the phagocytosis of bacteria [12]. Future in situ hybridisation work in our laboratory will target *Cat L*-like in the various haemocyte cells of *Btk*-poisoned insects to expand our understanding of the role of this cathepsin in defence against bacterial pathogens.

Pang et al., (2020) showed that *Cat L*-like expression peaks following injection of the steroid moulting hormone 20-hydroxyecdysone (20E) into silkworm larvae [16]. Overexpression of *Cat L*-like in response to *Btk* intoxication may also be triggered by 20E. Guo et al., (2020) linked high endogenous 20E titres and *Bt* resistance in the diamondback moth (*Plutella xylostella*) via a mechanism involving activation of the mitogen-activated protein kinase kinase kinase 4 (MAP4K4) signalling cascade [25]. Indeed, 20E— bound to its nuclear receptor (the EcR/USP complex)— regulates not only the expression of genes involved in developmental transition processes such as moulting and metamorphosis [26,27] but also that of metabolism and immunity [28,29]. An example of the regulation of insect immunity by 20E is *Anopheles gambiae*, the mosquito vector of *Plasmodium berghei* (a murine malaria species), in which 20E priming induced the expression of immune genes involved in pathogen defence and enhanced antibacterial and anti-*Plasmodium* immunity [30]. The existence of 20E regulatory elements for *Cat L*-like transcription has not yet been identified. However, Cheng et al., (2018) found that not all 20E-regulated silkworm genes contain ecdysone response element motifs and suggested that the activated EcR/USP receptor complex may directly or indirectly regulate target genes [31].

We successfully knocked down the *Cat L*-like gene in silkworm larvae using an RNAi approach and showed in bioassays that it is required for the insect’s survival response to the *Btk* pathogen, i.e., the RNAi larvae were more susceptible to the biopesticide Dipel. In addition, the RNAi insects were a more suitable host for *Btk* replication (when treated with Dipel 2 μg/mL, dsRNA-Cat L-like-insects produced a higher number of spores per cadaver compared with controls), suggesting that Cat L-like cathepsin may be involved in an antibacterial killing mechanism of haemocytes. However, no *Btk* proliferation was detected in the cadavers of insects exposed to the lowest Dipel dose tested (0.4 μg/mL), probably because other bacterial species in the silkworm gut flora participated in the heterospecific competition that severely limited *Btk* growth. The latter supports previous findings that under certain circumstances predominant symbiotic species of insect gut flora can invade the haemocoel through the *Bt*-damaged gut epithelium and exert pathogenic effects [32,33].

Cat L-like sequences are conserved in moths and the same is expected of their functions. Interestingly, phylogenetic analysis of Cat L-like showed that some of the homologous sequences that belong to moths are important plant pests, such as the fall armyworm *Spodoptera frugiperda,* a new invasive maize pest in Africa, Asia, and Oceania [34]. The fall armyworm has also developed *Bt* resistance in the past [35,36] and new targets and strategies for its control are constantly being sought. On the other hand, the potential orthologue of the silkworm Cat L-like cathepsin in *Helicoverpa armigera* (Acc. no. ACD40324) was previously found to be required for the completion of metamorphosis in this insect species via a mechanism not yet fully elucidated [37]. In our study, no significant differences in pupal formation were found between RNAi and control silkworms. However, we did not test the persistence of *Cat L*-like RNAi and whether the suppression of *Cat L*-like expression in the larval stage was still significant at the time of cocoon formation.

In summary, we have shown that silkworm Cat L-like cathepsin is required for defence against the *Btk* entomopathogen in this insect species, possibly through its involvement in the degradation of engulfed bacteria in haemocyte lysosomes. Accordingly, we propose that it could be used as a target for new pest management strategies to increase the insecticidal efficacy of *Bt*-based biopesticides.

## 4. Materials and Methods

### 4.1. Insect Strain

All experiments were carried out with a population of the inbreed silkworm strain Dazao (P50) from the Sericultural Research Institute (Chinese Academy of Agricultural Sciences). Insects were maintained in the Nanyang Normal University, reared at 25 °C ± 2 °C and ca. 60% relative humidity (RH) under 12-h: 12-h (light: dark) photoperiod on fresh white mulberry (*Morus alba* Linnaeus) leaves.

### 4.2. B. thuringiensis and Bioassays

Serial dilutions of a dry flowable (DF) formulation of the *Btk* strain ABTS 351-based biopesticide Dipel (32 000 International Units/mg) (Valent BioSciences, Libertyville, IL, USA) were freshly prepared using 0.1% Triton X-100 in water and used for standard leaf dip bioassay of toxicity [38]. Briefly, medium-sized mulberry leaves were dipped into Dipel dilutions (50 μg/mL, 10 μg/mL, 2 μg/mL, 0.4 μg/mL) for 30 s each and then laid flat on a non-absorbent plastic for one hour to air dry. Control leaves were treated with 0.1% Triton X-100 alone. Five one-day-old 5th instar silkworm larvae were confined on single leaf disks on moist filter paper in 100 mm Petri dishes, with six dishes per dose and control (30 larvae in all). Assay plates were incubated at 25 °C, 60% RH, and 12:12 (light/dark) cycle, and the 3-day mortality was recorded (i.e., dead larvae failed to respond after gentle prodding).

### 4.3. RNA Extraction and cDNA Synthesis

Total RNA from silkworm haemolymph was extracted using the Easy Pure RNA kit (TransGen Biotech, Beijing, China) and treated with DNase I (Promega, Madison, WI, USA). The concentration of total RNA was measured by spectrophotometry on NanoDrop 2000 (Thermo Scientific, Waltham, MA, USA). First-strand complementary DNA (cDNA) was synthesised from 2 μg of total RNA with a RevertAid first-strand cDNA synthesis kit (Thermo Fisher, Waltham, MA, USA). No reverse transcriptase controls were prepared to assess the level of contaminating genomic DNA. All procedures were carried out according to manufacturers’ instructions.

### 4.4. Quantitative RT-PCR (qRT-PCR) Experiments

Roche FastStart universal SYBR green master mix (Rox) (Roche, Basel, Switzerland) was used for 20-μL qRT-PCR reactions. Each reaction contained 10 μL of the 2x concentrated FastStart Universal SYBR Green master mix (ROX), 300 nM of each primer (*Cat L*-like-qRT-F, 5′-TCTTACACAAATGCGGACGG-3′; *Cat L*-like-qRT-R, 5′-GCAGCTACCTTCAAAGCCTCA-3′ [16]), and 50 ng of template cDNA. The reactions were performed in triplicate on a Bio-Rad CFX96 Touch real-time PCR system (Bio-Rad). The protocol followed a standard two-step PCR program consisting of polymerase activation at 95 °C for 3 min followed by 40 cycles of denaturation at 95 °C for 15 s and annealing/extension and read out at 60 °C for 30 s. The specificity was determined by post-PCR melt curve analysis at 55 °C to 95 °C. The glyceraldehyde-3-phosphate dehydrogenase (*GAPDH*) gene (GenBank accession no. XM_012690444) was used for transcript normalisation (*GAPDH*-qRT-F, 5′-CATTCCGCGTCCCTGTTGCTAAT-3′; *GAPDH*-qRT-R, 5′-GCTGCCTCCTTGACCTTTTGC-3′) [16]. The threshold cycle (CT) values were obtained from three independent experiments. The amplification efficiencies of *Cat L*-like and *GAPDH* genes were determined through standard curves (Table S1). The relative transcript levels were expressed as ‘Mean Normalized Expression’ data using Q-GENE software [39].

### 4.5. Synthesis of Double-Stranded RNA (dsRNA)

DNA templates from *Cat L*-like (GenBank accession no. AB436161) and *bla* (beta-lactamase gene in plasmid pUC19; GenBank accession no. M77789) were produced by PCR using mutagenic oligonucleotides elongated with the T7 promoter sequence. A 699-bp *Cat L*-like fragment was amplified with primers 5′-GGATCCTAATACGACTCACTATAGGCCAGCAGGATCCACAATCTT-3′ and 5′-GGATCCTAATACGACTCACTATAGGTACGTCGGACCAGATTCACA-3′, whereas a 567-bp *bla* amplicon was obtained with primers 5′-GGATCCTAATACGACTCACTATAGGAAGCCATACCAAACGACGAG-3′ and 5′-GGATCCTAATACGACTCACTATAGGTTTGCAAGCAGCAGATTACG-3′ (the part corresponding with the T7 promoter sequence appears underlined).

The synthesis of dsRNA-Cat L-like and dsRNA-bla (nonspecific dsRNA control) by in vitro transcription was performed using the T7 RiboMAX Express RNAi System (Promega) according to the manufacturer’s protocol.

### 4.6. dsRNA Injection for RNAi Induction

One hundred and twenty-five one-day-old 5th instar larvae were injected once into the base of an abdominal proleg with 1 μL of dsRNA-Cat L (2.5 μg/mL). Control insects were injected with the same volume of either nonspecific dsRNA-bla (2.5 μg/mL) or nuclease-free water (*n* = 125 each). Injected larvae were returned to the rearing cages and provided with mulberry leaves one hour after injection. Dipel bioassays of RNAi insects were conducted as above at 48 h post-injection.

### 4.7. Btk Reproduction within RNAi Insect Cadavers

Dead RNAi insects from the Dipel bioassays were individually crushed and homogenised in 1 mL of phosphate-buffered saline (PBS) (137 mM NaCl, 10 mM Na_2_HPO_4_, 1.8 mM KH_2_PO_4_, 2.7 mM KCl, pH 7.4) using a sterile glass pestle. Homogenates of cadavers were pasteurised (70 °C for 30 min) and used for preparing ten-fold serial dilutions in PBS. Triplicate 10 μL aliquots from each dilution were dropped onto LB agar using the track-dilution method [40] and plates were incubated at 30 °C for 24 h to generate a *Btk* spore count that was normalised to CFU/mL and converted to the equivalent log10 value. Homogenates of live larvae from the Triton X-100—exposed group were used as the negative control.

### 4.8. Phylogenetic Analysis

Multiple sequence alignments and phylogenetic analyses were performed using the NGPhylogeny.fr platform [41,42]. The deduced amino acid sequence of the Cat L-like protein was compared with the non-redundant GenBank and UniProtKB/TrEMBL protein databases and 34 closely related Lepidoptera sequences were selected for phylogenetic analysis. Bootstrap confidence levels were determined with 1000 replicates. Graphical representation and editing of the phylogenetic tree were performed using iTOL version 6.5.4 [43,44].

### 4.9. Statistical Analysis

Student’s *t*-tests and one-way analysis of variance (ANOVA) were performed using GraphPad Prism software version 8.0.2 for Windows. The analysis of bioassays, including larval mortality in the RNAi experiments, was carried out using the open-source R environment [45]. Estimates of LC_50_, 95% Fiducial Limits, and slopes were calculated by maximum likelihood logit regression analysis in a generalized linear model from individually fitted analyses of deviance. The quasibinomial was used to adjust for overdispersion. Specific *post hoc* comparisons between levels within treatments were performed using model treatment contrasts. The experiments were performed three times with three independent replicates per treatment to assess reproducibility. However, the data from one representative experiment are shown. The replicates were presented as mean values ± standard error of the mean.

## Data Availability

The data presented in this study are available in the article.

## Supplementary Materials

The following supporting information can be downloaded at: https://www.mdpi.com/article/10.3390/toxins14060394/s1, Table S1: Experimental conditions used in quantitative RT-PCR.
